# A122 INTEROBSERVER AGREEMENT FOR THE PARIS CLASSIFICATION OF COLORECTAL POLYPS AMONGST SURGEONS, GASTROENTEROLOGISTS, TRAINEES AND EXPERTS: A VIDEO-BASED STUDY

**DOI:** 10.1093/jcag/gwad061.122

**Published:** 2024-02-14

**Authors:** A Alj, R Djinbachian, M Pioche, Y Saito, A Sethi, P Chiu, J Marks, H Sebajang, K Pawlak, A Chennouf, F Benard, Y Ide, F Dang, D von Renteln

**Affiliations:** CRCHUM, Montréal, QC, Canada; CRCHUM, Montréal, QC, Canada; CRCHUM, Montréal, QC, Canada; CRCHUM, Montréal, QC, Canada; CRCHUM, Montréal, QC, Canada; CRCHUM, Montréal, QC, Canada; CRCHUM, Montréal, QC, Canada; CRCHUM, Montréal, QC, Canada; CRCHUM, Montréal, QC, Canada; CRCHUM, Montréal, QC, Canada; CRCHUM, Montréal, QC, Canada; CRCHUM, Montréal, QC, Canada; CRCHUM, Montréal, QC, Canada; CRCHUM, Montréal, QC, Canada

## Abstract

**Background:**

Colonic polyps are described based on their macroscopic shape, according to the Paris classification. This represents a useful tool to communicate polyp morphology between endoscopists, however, the agreement between endoscopists on polyp morphology evaluation has not been demonstrated in the literature. We were interested in evaluating endoscopist agreement using the Paris classification.

**Aims:**

The aim of this study was to evaluate endoscopist agreement using the Paris classification.

**Methods:**

We conducted a prospective video-based study evaluating endoscopist assessment of polyp morphology. An international group of endoscopists including surgeons, gastroenterologists, experts and trainees were included. Primary outcome was overall inter-observer agreement for the Paris classification. Secondary outcomes were inter-observer agreement stratified by endoscopist characteristics.

**Results:**

11 endoscopists were included in the analysis with 1320 morphologic assessments of 120 individual polyps. There was low overall inter-observer agreement for the Paris classification with an overall Light’s Kappa of 0.19. Agreement remained low for men (Κ=0.25), women (Κ=0.25), gastroenterologists (K=0.23), surgeons (K=0.11), trainees (K=0.33), and experts (K=0.29). Endoscopists who have received training on lesion evaluation in Asia had higher inter-observer agreement when using the Paris classification compared to endoscopists in the west (K= 0.50 vs 0.26).

**Conclusions:**

Agreement when using the Paris classification was low amongst a varied international group of surgeons, gastroenterologists, trainees and experts. Training in the east where higher emphasis is placed on lesion characterization was associated with the highest inter-observer agreement.

**Funding Agencies:**

None

